# Long QT Syndrome: An Emerging Role for Inflammation and Immunity

**DOI:** 10.3389/fcvm.2015.00026

**Published:** 2015-05-27

**Authors:** Pietro Enea Lazzerini, Pier Leopoldo Capecchi, Franco Laghi-Pasini

**Affiliations:** ^1^Department of Medical Sciences, Surgery and Neurosciences, University of Siena, Siena, Italy

**Keywords:** long QT syndrome, inflammation, cytokines, immunity, autoantibodies, anti-Ro/SSA

## Abstract

The long QT syndrome (LQTS), classified as congenital or acquired, is a multi-factorial disorder of myocardial repolarization predisposing to life-threatening ventricular arrhythmias, particularly torsades de pointes. In the latest years, inflammation and immunity have been increasingly recognized as novel factors crucially involved in modulating ventricular repolarization. In the present paper, we critically review the available information on this topic, also analyzing putative mechanisms and potential interplays with the other etiologic factors, either acquired or inherited. Accumulating data indicate inflammatory activation as a potential cause of acquired LQTS. The putative underlying mechanisms are complex but essentially cytokine-mediated, including both direct actions on cardiomyocyte ion channels expression and function, and indirect effects resulting from an increased central nervous system sympathetic drive on the heart. Autoimmunity represents another recently arising cause of acquired LQTS. Indeed, increasing evidence demonstrates that autoantibodies may affect myocardial electric properties by directly cross-reacting with the cardiomyocyte and interfering with specific ion currents as a result of molecular mimicry mechanisms. Intriguingly, recent data suggest that inflammation and immunity may be also involved in modulating the clinical expression of congenital forms of LQTS, possibly triggering or enhancing electrical instability in patients who already are genetically predisposed to arrhythmias. In this view, targeting immuno-inflammatory pathways may in the future represent an attractive therapeutic approach in a number of LQTS patients, thus opening new exciting avenues in antiarrhythmic therapy.

## Introduction

**Figure 1:**
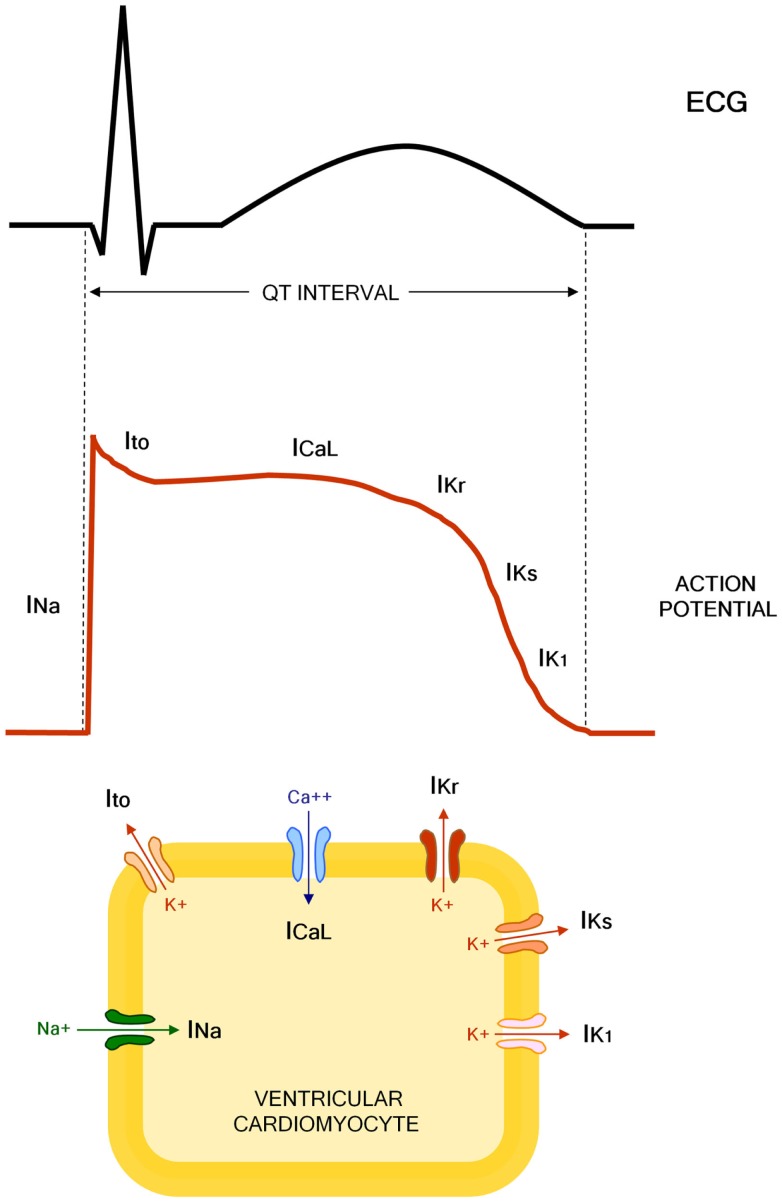
**Molecular and electrophysiological basis of QT interval**. INa, sodium current; Ito, transient outward current; ICaL, L(long-lasting)-type calcium current; IKr, rapid component of the delayed rectifier potassium current; IKs, slow component of the delayed rectifier potassium current; IK1, inward rectifier potassium current.

The QT interval indicates the duration of action potential (AP) in ventricles, which represents the sum of ventricular depolarization and repolarization. AP is caused by transmembrane flow of ions, including inward depolarizing currents mainly through sodium and calcium channels, and outward repolarizing currents mainly through potassium channels. More in details, six sequentially activated currents are fundamentally involved: the sodium current (INa), the transient outward current (Ito), the L(long-lasting)-type calcium current (ICaL), the rapid component of the delayed rectifier potassium current (IKr), the slow component of the delayed rectifier potassium current (IKs), and the inward rectifier potassium current (IK1) (Figure 1).

The Long QT Syndrome (LQTS) is a multi-factorial disorder of myocardial repolarization characterized by a prolonged corrected QT interval (QTc) on the electrocardiogram (ECG), and predisposing to life-threatening ventricular arrhythmias, particularly torsades de pointes (TdP) (1). The LQTS is traditionally classified as congenital or acquired (1, 2), even though it has becoming clear how in many cases the clinical phenotype is the result of a complex interaction of multiple etiologic factors operating concomitantly in the single patient (3).

Congenital LQTS, which can often be a lethal disorder (2), is caused by genetically determined abnormalities affecting directly or indirectly the function of specific ionic channels involved in ventricular AP, i.e., potassium (loss of function), sodium or calcium channels (gain of function) (4). To date, about 1000 mutations in 13 LQTS-susceptibility genes have been identified; however, only three of these genes, namely KCNQ1 (encoding KvLQT1 channel α-subunit, conducting IKs), KCNH2 (encoding hERG channel α-subunit, conducting IKr), and SCN5A (encoding Nav1.5 channel α-subunit, conducting INa), account by themselves for ~75% of all cases (5). The incidence of the congenital LQTS is not well known, although a recent clinical-genetic analysis on ~45,000 neonates suggests that it may be close to 1:2000 live births (6).

Acquired LQTS is much more prevalent than the congenital form, although its precise incidence and mortality impact in the general population are difficult to be estimated. Nevertheless, recent studies demonstrated that QTc prolongation is highly prevalent (up to 25–30% of hospitalized patients), also preliminarly suggesting that acquired LQTS may be as risky as congenital LQTS (7–10). More frequently, acquired LQTS represents an adverse effect of drugs or the result of electrolyte disturbances interfering with cardiomyocyte electrophysiology (1). In particular, the molecular basis of drug-induced LQTS almost exclusively involves the reduction of IKr through hERG-potassium channel blockade (11). Other currently recognized causes of acquired LQTS include structural heart diseases, bradyarrhythmias, endocrine disorders, liver diseases, nervous system injuries, HIV infection, starvation, hypothermia, and toxins (1, 12–14).

In the latest years, mounting evidence from basic and clinical studies strongly suggests that inflammation and immunity represent further important determinants of acquired LQTS. In the present paper, we review the available data on this topic, also analyzing putative mechanisms and potential interplays with the other etiologic factors, either acquired and inherited.

A list of the causes of acquired LQTS, also including inflammatory- and immune-mediated forms, is proposed in Table 1.

**Table 1:** **Causes of acquired long QT syndrome**.

**1. Drugs**
-Antiarrhythmic drugs (class I and class III)
-Antimicrobials (fluoroquinolones, macrolides, imidazole antifungals, antimalarials, HIV protease inhibitors)
-Antihistamines (histamine H1-receptor antagonists)
-Psychoactive agents (antidepressants, antipsychotics, lithium, methadone)
-Motility and antiemetic drugs (cisapride, domperidone, serotonin 5-HT3-receptor antagonists)
-Anticancer drugs (arsenic trioxide, tamoxifen)
-Diuretics (indapamide)
-Inotropics (phosphodiesterase III inhibitors)
-Immunosuppressants (tacrolimus)
**2. Electrolyte imbalances**
-Hypokalemia, hypocalcemia, hypomagnesemia
**3. Structural heart diseases**
-Ischemic heart disease, left ventricular hypertrophy, heart failure, Takotsubo cardiomyopathy
**4. Bradyarrhythmias**
-Complete atrioventricular block (or any bradyarrhythmia, even transient)
**5. Endocrine disorders**
-Hypothyroidism, corticosteroid insufficiency, diabetes mellitus, pheocrhromocytoma
**6. Inflammatory diseases**
-Inflammatory heart diseases (myocarditis, Chagas’s disease, rheumatic heart disease)
-Systemic inflammatory diseases (rheumatoid arthritis, connective tissue diseases)
**7. Autoimmunity**
-Anti-Ro/SSA antibodies
-Other autoantibodies (anti−β1-adrenergic receptor, anti-Kv1.4 potassium channel)
**8. End-stage liver disease**
**9. Nervous system injuries**
-Subarachnoid hemorrhage, thalamic hematoma, right neck dissection, autonomic neuropathy
**10. HIV infection**
**11. Starvation**
-Anorexia nervosa, “liquid protein” diets, gastroplasty and ileojejunal bypass, celiac disease
**12. Hypothermia**
**13. Toxins**
-Cocaine, arsenic, organophosphates (insecticides, nerve gas)

## Inflammation as a Cause of Acquired LQTS

### Clinical data

Several lines of evidence support the hypothesis that inflammation, either cardiac or systemic, significantly impacts on QT interval duration and related risk of life-threatening arrhythmias (Table 2).

**Table 2:** **Inflammation and QTc prolongation: clinical studies**.

Reference	Study population	Subjects	Controls	Main findings
		*n*	*n*	
**INFLAMMATORY HEART DISEASES**				
Ramamurthy et al. (15)	Myocarditis (biopsy-proven)	20	–	QTc prolongation was the most common ECG abnormality (70%)
Ukena et al. (16)	Myocarditis	186	–	QTc prolongation (25% of patients) predicted cardiac death
Williams-Blangero et al. (17)	Chagas’ disease	722	667	Mean QT intervals longer in *T. Cruzi* seropositive than seronegative subjects
Salles et al. (18)	Chagas’ disease	738	–	QTc max was an independent predictor of sudden death
Santos et al. (19)	Acute rheumatic carditis	27	–	QTc prolongation was the most common ECG abnormality (30%)
Balli et al. (20)	Acute rheumatic carditis	73	–	A prolonged QTc correlated with both presence of carditis and levels of acute phase reactants
**SYSTEMIC INFLAMMATORY DISEASES**				
Lazzerini et al. (21)	Rheumatoid arthritis	25	20	Mean QTc longer in RA patients than healthy controls and correlated with CRP levels
Chauhan et al. (22)	Rheumatoid arthritis	518	499	Cumulative incidence of QTc prolongation higher in RA than non-RA patients; any QTc prolongation independently associated with all-cause mortality; idiopathic QTc prolongation correlated with ESR
Panoulas et al. (23)	Rheumatoid arthritis	357	–	QTc prolongation was independently associated with CRP levels and predicted all-cause mortality
Adlan et al. (24)	Rheumatoid arthritis	112	–	QTc prolongation correlated with circulating levels of inflammatory cytokines
Lazzerini et al. (25)	Rheumatoid arthritis	17	–	Anti-IL-6 therapy (TCZ) was associated with a rapid QTc shortening, which correlated with the decrease in both CRP and TNFα levels
Lazzerini et al. (26)	Connective tissue diseases	57	–	QTc prolongation in 31% of patients
Costedoat-Chalumeau et al. (27)	Connective tissue diseases	89	–	QTc prolongation in 12% of patients
Lazzerini et al. (28)	Connective tissue diseases	46	–	QTc prolongation (28% of patients) correlated with complex ventricular arrhythmias
Lazzerini et al. (29)	Connective tissue diseases	49	–	QTc prolongation in 32% of patients
Pisoni et al. (30)	Connective tissue diseases	73	–	QTc prolongation (15% of patients) was independently predicted by circulating IL-1β levels
Cardoso et al. (31)	Systemic lupus erythematosus	140	37	Mean QTc longer in SLE patients than healthy controls
Milovanović et al. (32)	systemic lupus erythematosus	52	41	Mean QTc longer in SLE patients than healthy controls
Bourrè-Tessier et al. (33)	Systemic lupus erythematosus (two studies)	150	–	QTc prolongation (7% of patients) was independently associated with SDI
		278	–	
Bourrè-Tessier et al. (34)	Systemic lupus erythematosus	779	–	QTc prolongation (15% of patients) was independently associated with SDI
Alkmim Teixera et al. (35)	Systemic lupus erythematosus	317	–	Marked QTc prolongation (>500 ms) in 3% of patients
Sgreccia et al. (36)	Systemic sclerosis	38	17	Mean QTc was longer in SSc patients than healthy controls
Massie et al. (37)	Systemic sclerosis	689	–	QTc prolongation (25% of patients) was independently associated with disease duration and severity
**NON-INFLAMMATORY HEART DISEASES**				
Chang et al. (38)	Arterial hypertension	466	–	CRP levels correlated with QTc duration and independently predicted QTc prolongation
Yue et al. (39)	Coronary artery disease	56	–	CRP levels correlated with QTc duration
Song et al. (40)	Takotsubo cardiomyopathy	105	–	Patients with QTc prolongation had higher CRP levels than those with normal QTc
**GENERAL POPULATION**				
Kazumi et al. (41)	Healthy subjects	179	–	QTc length independently correlated with CRP
Kim et al. (42)	Healthy subjects	4758	–	QTc prolondation independently associated with elevated CRP
Medenwald et al. (43)	Healthy subjects	1716	–	Soluble TNF-receptor 1 levels independently correlated with QTc duration in women

*ECG, electrocardiogram; QTc, corrected QT interval; RA, rheumatoid arthritis; CRP, C-reactive protein; ESR, erythrocyte sedimentation rate; TCZ, tocilizumab; CTD, connective tissue disease; SLE, systemic lupus erythematosus; SSc, systemic sclerosis; TNF-α levels, tumor necrosis factor alpha; IL-1β, interleukin-1 beta; SDI, Systemic Lupus International Collaborating Clinics/American College of Rheumatology (SLICC/ACR) damage index*.

First of all, inflammatory heart diseases, particularly myocarditis, are frequently associated with QTc prolongation, in some cases of severe degree. Indeed, in myocarditis patients, a prolonged QTc was found to be the most common electrocardiographic abnormality observed (15), also associating with the occurrence of complex ventricular arrhythmias (44). Accordingly, in a large cohort of 186 patients with myocarditis, Ukena et al. (16) demonstrated that a QTc prolongation ≥440 ms was frequent (~25% of cases) and predicted a poor clinical outcome, including cardiac death. Moreover, several cases of marked QTc prolongation complicated with TdP have been reported in patients with acute infective carditis (myo/endocarditis), independently from the specific etiologic agent involved (45–57). A peculiar form of diffuse myocarditis is Chagas’s disease, triggered by the protozoan *Trypanosoma cruzi*, and then progressing for (auto)immune-mediated mechanisms (58). In this form, QTc prolongation represents a common and prognostically relevant feature too (17, 18). Noteworthy, in murine models of the disease, a significant correlation between QTc duration and the degree of cardiac inflammation at the histological examination has been demonstrated (59, 60). Finally, evidence also exists that QTc is often prolonged in a purely immune-mediated inflammatory heart disease such as acute rheumatic carditis (61), with some cases even complicating with TdP (62, 63). In particular, an increased QTc has been found to be the most frequent ECG alteration in children with subclinical carditis (~30%) (19). Moreover, in patients with acute rheumatic fever, a prolonged QTc correlated with both the presence of carditis and the level of acute-phase reactants (20).

Not only cardiac, but also systemic inflammation is associated with QT prolongation, as indicated by accumulating data obtained in patients with autoimmune chronic inflammatory diseases, as well as in patients affected with non-inflammatory heart diseases or apparently healthy subjects from general population.

Among systemic autoimmune diseases, the largest evidence regards rheumatoid arthritis (RA) and connective tissue diseases (CTDs). In RA, representing a paradigmatic example of chronic disease with high-grade inflammatory burden, the risk of sudden cardiac death (SCD) is approximately two times higher than in non-RA subjects (64). Recent studies demonstrated that in RA patients, QTc is frequently prolonged, associates with disease severity and inflammatory markers, and predicts mortality (65). In a cohort of 101 patients with chronic inflammatory arthritis, in which a significant positive correlation between C-reactive protein (CRP) values and QTc duration was demonstrated, we found that RA patients had a longer QTc when compared with both spondyloarthritis patients and healthy controls (21). These findings were very recently confirmed in a larger retrospective, population-based cohort study involving 650 RA patients and 650 age- and sex-matched non-RA patients. During follow-up, the cumulative incidence of QTc prolongation at 20 years after RA incidence (or after index date for controls) was higher among RA than non-RA subjects. Notably, in RA patients, erythrocyte sedimentation rate (ESR) at diagnosis was significantly associated with risk of idiopathic QTc prolongation, i.e., excluding prolongations explained by ECG changes, medications, etc. (22). In another prospective study carried out on 357 RA patients, it was found that prolonged QTc is a strong predictor of death as a 50 ms increase in QTc interval associated with a doubling of the hazard for all-cause mortality (22). The evidence that in this population, QTc prolongation was independently associated with CRP levels, and that the significance of the association between QTc and all-cause mortality was lost after the adjustment for CRP, once more and robustly supported the hypothesis that systemic inflammation plays a key mechanistic role in the phenomenon. As a further confirmation of this view, Adlan et al. (24) found that in RA patients circulating levels of inflammatory cytokines (TNFα, IL-1β, IL-6, IL-10) correlated with QTc duration. Moreover, we demonstrated that in RA anti-cytokine therapy with the anti-interleukin 6-receptor antibody, tocilizumab was associated with a rapid and significant QTc shortening, which correlated throughout the study time with the decrease in both CRP, and, more strongly, circulating TNFα levels (25).

Several studies performed in patients with different CTDs reported a high overall prevalence of QTc prolongation (up to ~30%) (26–30), with circulating IL-1β levels independently predicting the presence of a prolonged QTc (30). As regards specific CTD forms, it has been demonstrated that systemic lupus erythematosus (SLE) patients display longer mean QTc than controls (31, 32), and data obtained from large SLE cohorts found a 7–15% incidence of QTc prolongation (33, 34) [marked QTc prolongation, i.e., >500 ms, in ~3% (35)], with a significant association between QTc and overall inflammatory burden, as reflected by SLICC/ACR damage index (SDI) (34, 35). Noteworthy, 10 cases of drug-induced TdP in SLE patients were reported (66–75), and although CRP was specifically assessed only in two cases, nevertheless it was elevated in both (70, 74). Maximum QTc is also increased in systemic sclerosis (SSc) patients when compared to healthy controls (36). Furthermore, a recent study on 689 SSc patients demonstrated that QTc prolongation occurs in 25% of the cases, also independently correlating with disease duration and disease severity (37). Finally, preliminary results suggest that an increased frequency of QTc prolongation may be observed in other chronic inflammatory diseases, particularly inflammatory bowel disease and psoriasis (76, 77).

Systemic inflammation can also be involved in the pathogenesis of QTc prolongation in some non-inflammatory heart diseases. By analyzing 466 hypertensive patients, Chang et al. (38) found that CRP level correlated with QTc and independently predicted QTc prolongation presence. Similarly, in patients with coronary artery disease, a significant association between QTc duration and circulating CRP was observed (39). Moreover, a study involving 105 patients with Takotsubo cardiomyopathy demonstrated that subjects presented with QTc prolongation had higher CRP levels than those with normal QTc (40).

Finally, a significant relationship between the degree of systemic inflammatory activation and QTc duration is also observed in apparently healthy subjects in general population. The first report by Kazumi et al. (41) showed that QTc length was independently correlated with CRP in 179 males aged 18–22 years. More recently, two large population-based studies involving middle-aged or elderly subjects confirmed these findings. In the first one, Kim et al. (42) analyzed 4758 individuals (40–69 years) and concluded that prolonged QTc was significantly associated with elevated CRP (about twofold increase in the odds of QTc being ≥440 ms), independent of confunders. Accordingly, in the CARdiovascular diseases, Living, and Aging in Halle (CARLA) Study involving 1716 subjects aged 45–83 years, parameters of inflammation correlated with QTc duration, particularly soluble TNF-receptor-1 levels (sTNF-R1, a circulating stabile marker of TNFα system activation) in women (43). The concomitant evidence from large prospective community-based studies that inflammatory markers (CRP, IL-6) predict SCD in apparently healthy persons (78, 79) suggests that this association, at least in part, may be explained by a higher propensity to develop long QT-associated malignant arrhythmias.

### Mechanisms

**Figure 2:**
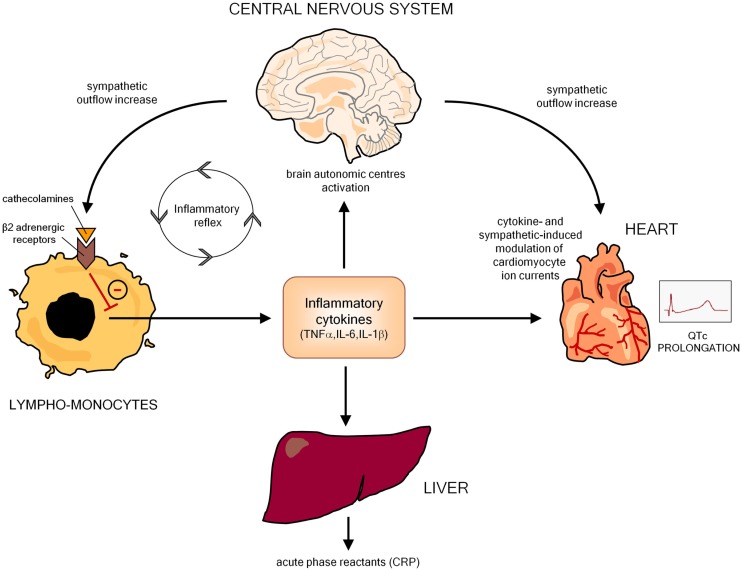
**Potential mechanisms responsible for inflammation-mediated QTc prolongation**. CRP, C-reactive protein.

An increasing body of evidence indicates that inflammatory activation profoundly impacts the electrophysiological properties of cardiomyocytes via multiple effects, ultimately resulting in a prolongation of AP duration (APD), and thereby of the QTc on ECG. In this scenario, the key mediators seem to be inflammatory cytokines (particularly TNFα, IL-6, IL-1β), which may affect myocardium either directly, by modulating specific ion channels critically involved in APD, and indirectly, by increasing central nervous system sympathetic drive on the heart (Figure 2).

A number of basic studies demonstrated significant direct effects of inflammatory cytokines on cardiac electrophysiology, particularly inducing changes in the expression and function of potassium and calcium channels (Table 3). Perfused hearts from transgenic mice overexpressing TNFα exhibited a prolonged APD and re-entrant ventricular arrhythmias (80); left ventricular myocytes isolated from these animals revealed a robust decrease of Ito and a reduced expression of the corresponding potassium channel protein (81). Several authors reported consistent findings when rat ventricular myocytes were cultured with TNFα, also demonstrating the involvement of a molecular cascade including iNOS overexpression, oxidant species generation, NFκB activation, and potassium-channel-interacting protein 2 (KChIP-2) inhibition (82–84). Moreover, Wang and coll (85) showed that TNFα down-regulates *in vitro* IKr by impairing the function of the hERG potassium channel via the stimulation of reactive oxygen species. Although it is far probable that similar effects on potassium channels are also exerted by the other main pro-inflammatory cytokines IL-6 and IL-1, no specific studies evaluated this topic as yet. Nevertheless, experiments on pig and mouse ventricular cells clearly demonstrated the ability of both these cytokines to prolong APD, possibly by enhancing ICaL (86, 87). Finally, no data exist about possible effects of cytokines on sodium channels. This area needs further evaluation, given that an increase in the INa current may theoretically contribute to cytokine-induced APD prolongation. Although not fully elucidated, the previously reported evidence that circulating inflammatory cytokine levels correlated with QTc duration in patients with RA (24, 25), CTDs (30), as well as in healthy subjects (43) strongly indicate that also *in vivo*, these pathophysiological mechanisms are of crucial importance.

**Table 3:** **Effects of inflammatory cytokines on cardiomyocyte action potential: electrophysiological and molecular mechanisms**.

Cytokine	Effects on cardiomyocyte ion currents	Molecular mechanisms	Effect on APD
TNFα	IKr decrease (85)	Impairment of hERG potassium channel function (via stimulation of ROS) (85)	Prolongation (80, 83, 85)
	Ito decrease (81–83)	reduced expression of Kv4.2 and Kv4.3 potassium channels (81–83) [via iNOS induction (83), ROS generation (83), NKkB activation (84), and KChIP-2 inhibition (82, 84)]	
IL-1β	ICaL increase (86)	Lipoxygenase pathway-mediated (86)	Prolongation (86)
IL-6	ICaL increase (87)	Enhancement of Cav1.2 calcium channel function (via SHP2/ERK-mediated phosphorilation) (87)	Prolongation (87)

*TNFα, tumor necrosis factor α; IL-1β, interleukin-1β; IL-6, interleukin-6; ROS, reactive oxygen species; iNOS, inducible nitric oxyde synthase; NFkB, nuclear factor-kappa-B; KChIP-2, K(+) channel-interacting protein; SHP/ERK, Src homology 2 domain-containing phosphatase/extracellular signal-regulated kinase; APD, action potential duration*.

Animal models of cardiac or systemic inflammation confirm and expand the relevance of these data. In isolated ventricular myocytes from mice with experimental autoimmune myocarditis (EAM), APD was markedly prolonged and Ito density significantly reduced when compared to controls (88). An increased APD (with a higher susceptibility to triggered ventricular arrhythmias) was consistently reported by Park et al. (89) by analyzing EAM rat hearts in which an elevated tissue expression of IL-6 and TNFα was demonstrated. Notably, the authors also proved that both cytokine myocardial accumulation and electrophysiological changes were prevented by prednisone administration. Similar preventive effects, associated with a significant attenuation of Ito inhibition, were also reported by Tang et al. (90) by treating the animals with statins (atorvastatin), whose anti-inflammatory potential in myocarditis has being increasingly recognized (91). Moreover, relevant cardiac electrophysiological alterations have been recently demonstrated in a murine model of myocardial infarction (MI) in which a state of systemic inflammation was induced. In MI mice, intraperitoneal injection with lipopolysaccharide (LPS) was associated with repolarization and APD prolongation, and higher ventricular arrhythmia propensity than non-LPS-injected animals. Notably, LPS-treated mice showed increased inflammatory macrophage activity transmurally in the heart, with a strong relationship between the degree of local myocardial inflammation and electric remodeling. Furthermore, the authors provided indirect evidence of a link between electrophysiological abnormalities and higher IL-1β expression in the myocardium (92). Besides inducing macrophage-derived cytokine production, LPS may also prolong APD by directly downregulating Ito via toll-like receptor 4 activation, as recently demonstrated in isolated rat ventricular myocytes (93).

Inflammation can also produce cardiac electrophysiology changes leading to QTc prolongation in an indirect manner, by inducing autonomic nervous system (ANS) dysfunction. Indeed, many basic and clinical studies demonstrated that, by targeting the autonomic centers of the brain, inflammatory cytokines increase the sympathetic outflow overdrive that in turn inhibits cytokine production and immuno-inflammatory activation by stimulating the β2-adrenergic receptors expressed in circulating lymphocytes and monocytes. Such a self-controlling loop is a crucial component of the so-called *inflammatory reflex*, and in this context sympathetic activation putatively represents an adaptive response to damping the immuno-inflammatory response (94–97). However, central sympathetic system, when activated, affects not only the immune system, but also all the body districts under its control, including the heart, with relevant electrophysiological consequences on the myocardium (Figure 2). Indeed, cardiomyocyte β-adrenergic receptor activation profoundly and complexly affects calcium (ICaL) and potassium (IKs, IKr) conductance with a net effect of increase in APD (98). Accordingly, cardiac sympathetic denervation shortens APD in rats (99). Moreover, increased catecholamine levels typically prolong QTc in healthy individuals (100–102), and intravenous adrenaline produces increase in QTc length in congenital LQTS patients (103).

A large body of evidence demonstrates a strict relationship between the degree and duration of inflammatory activation and the severity of ANS dysfunction. In particular, many data focused on heart rate variability (HRV), a non-invasive method to detect early cardiovascular autonomic impairment by assessing the effects of the sympatho-vagal balance on the heart (104). Reduced HRV, indicating an increase in sympathetic and a reduction in parasympathetic nervous system activity (104), is a common finding in several systemic inflammatory immuno-mediated diseases, including chronic inflammatory arthritis (21, 65, 105, 106) and CTDs (26, 105), as well as in heart inflammatory diseases, including viral myocarditis (107) and acute rheumatic fever (108). Moreover, HRV parameters inversely correlated with circulating CRP (and/or inflammatory cytokines) in healthy individuals as well as in patients with cardiovascular diseases (109, 110). The amount of data on this topic available in RA is of particular significance. In these patients, cardiac ANS dysfunction is highly prevalent (~60%) with a main pattern indicative of elevated sympathetic activity and reduced parasympathetic activity (65, 106). Autonomic impairment (particularly HRV) associated with disease duration, disease activity, or inflammatory markers (65), and treatment with infliximab (a TNFα-antagonist monoclonal antibody) produced rapid and evident HRV changes, i.e., decrease in the sympathetic tone with a shift toward a relative vagal prevalence (97). Noteworthy, in patients with chronic inflammatory arthritis, systemic inflammation degree, as assessed by CRP, and HRV depression severity significantly correlated one each other and both with QTc duration (21).

## Autoimmunity as a Cause of Acquired LQTS

### Clinical data

In the last years, accumulating evidence indicates that autoimmune mechanisms are involved in the pathogenesis of cardiac arrhythmias (111). Indeed, a number of autoantibodies can deeply interfere with the bioelectric properties of the heart by directly targeting specific receptors, ion channels, or enzymes expressed on the cardiomyocyte surface (112, 113). In particular, increasing data demonstrated that some of these autoantibodies can increase the arrhythmic risk by inducing an acquired LQTS of autoimmune origin. Although most studies relate to anti-Ro/SSA antibodies, some data suggest that other autoantibodies may lead to QTc prolongation and related arrhythmias.

Anti-Ro/SSA antibodies (anti-Ro/SSA) consist of two fundamental subtypes, i.e., anti-Ro/SSA-52kD and anti-Ro/SSA-60kD, whose detection is frequent in CTDs, particularly Sjögren’s syndrome (30–95%) and SLE (30–50%), but also in 0.5–2.7% of the apparently healthy population (114). Large evidence links the trans-placental passage of anti-Ro/SSA from mother to fetus with the risk of developing congenital atrioventricular block (AVB) (115). Although traditionally considered as invulnerable, recent data suggest that also the adult conduction system may be affected by these antibodies (116). Moreover, increasing data indicate that anti-Ro/SSA significantly interfere with ventricular repolarization and promote QTc prolongation (114) (Table 4). In 2000, Cimaz et al. (117) for the first time reported a high prevalence (42%) of prolonged QTc in anti-Ro/SSA-positive infants without congenital-AVB. Later on, the same investigators demonstrated a concomitant disappearance of ECG abnormality and acquired maternal autoantibodies during their first year of life (118). Moreover, Gordon et al. (119) found that the QTc was significantly longer in children of anti-Ro/SSA-positive mothers compared with children of negative mothers, with a further increase in those with siblings with congenital-AVB. Consistent findings were obtained by several following studies performed in adults. In a cohort of adult CTD patients, we found that more than 50% of anti-Ro/SSA-positive subjects displayed a prolonged QTc, with mean QTc values significantly longer in positive vs negative patients (26). Accordingly, a similar prevalence of anti-Ro/SSA-associated QTc prolongation (46%) was demonstrated in a further 24-h ECG monitoring study on 46 CTD patients also showing that this ECG abnormality was associated with the occurrence of complex ventricular arrhythmias (28). More recently, Bourré-Tessier et al. (33) performed two consecutive large studies on 150 and 278 SLE patients, respectively. The authors found a 5.1- to 12.6-times higher risk of QTc prolongation in anti-Ro/SSA-positive group than in negative patients, and each 10 U/ml increase in anti-Ro/SSA titer was associated with a parallel increase in the risk of having prolonged QTc. The existence of a strict relationship between QTc length and antibody levels, as well as subtype specificity, was confirmed in a further study on 49 CTD patients performed in our institution. In this cohort, it was demonstrated a direct correlation between anti-Ro/SSA concentration and QTc duration, but with the anti-Ro/SSA-52kD subtype only when the two subtypes were considered separately (29). Very recently, Pisoni et al. (30) reported that among 73 CTD patients, 20% of anti-Ro/SSA-positive vs 0% of anti-Ro/SSA-negative subjects had QTc prolongation. Notably, in patients with prolonged QTc (all anti-Ro/SSA-positive), IL-1β levels were significantly higher than patients with normal QTc, thus intriguingly suggesting a synergistic interplay between autoantibodies and inflammatory cytokines on QTc duration. Furthermore, Nakamura et al. (120) described the case of a anti-Ro/SSA-positive woman with severe QTc prolongation and TdP in which clear evidence of a direct mechanistic link between circulating antibodies and QTc prolongation was provided (see Mechanisms). In this patient, no genetic or acquired known causes of QT prolongation were detected, although a polymorphism (D85N) in KCNE1 gene was found. Noteworthy, she was totally asymptomatic for autoimmune diseases. Since anti-Ro/SSA is the most frequent autoantibody found in general population, but in most cases (60–70%) totally asymptomatic (114), an intriguing speculation is that by reducing the repolarization reserve anti-Ro/SSA may be silently involved as a predisposing factor in a number of “idiopathic” life-threatening arrhythmias, including drug-induced TdP, and sudden unexpected deaths occurring in apparently healthy people.

**Table 4:** **Clinical studies on anti-Ro/SSA antibodies and QTc interval**.

Reference	Study population	Anti-Ro/SSA+ patients (*n*)	Anti-Ro/SSA− patients (*n*)	Main findings
Cimaz et al. (117)	Children of CTD mothers	21	7	Mean QTc significantly longer in anti-Ro/SSA-positive subjects
Gordon et al. (119)	Children of CTD mothers	38	7	Mean QTc significantly longer in children of anti-Ro/SSA-positive mothers
Gordon et al. (121)	Adult AD patients	49 (SLE, 29; SS, 11; other ADs, 9)	62 (SLE, 48; SS, 2; other ADs, 12)	Mean QTc slightly longer in anti-Ro/SSA-positive patients (*p* = 0.06)
Cimaz et al. (118)	Children of CTD mothers	21	–	Concomitant disappearance of QTc prolongation and acquired maternal antibodies at 1-year follow-up
Lazzerini et al. (122)	Adult CTD patients	31 (SLE, 6; SS, 14; SSc, 4; UCTD, 5; MCTD, 1)	26 (SLE, 4; SS, 1; SSc, 17; UCTD, 3; MCTD, 1)	Mean QTc significantly longer and prevalence of QTc prolongation significantly higher in anti-Ro/SSA-positive subjects
Costedoat-Chalumeau et al. (123)	Children of CTD mothers	58	85	No differences in mean QTc duration or in QTc prolongation prevalence between groups
Costedoat-Chalumeau et al. (27)	Adult CTD patients	32 (SLE, 28; SS, 4)	57 (SLE, 49; UCTD, 4; MCTD, 4)	No differences in mean QTc duration or in QTc prolongation prevalence between groups
Lazzerini et al. (28)	Adult CTD patients	26 (SLE, 4; SS, 9; SSc, 2; UCTD, 8; MCTD, 2; PM/DM, 1)	20 (SLE, 9; SS, 3; SSc, 4; UCTD, 1; MCTD, 2; PM/DM, 1)	Mean QTc significantly longer and prevalence of QTc prolongation significantly higher in anti-Ro/SSA-positive subjects; QTc prolongation significantly associated with the presence of complex ventricular arrhythmias
Motta et al. (124)	Children of CTD mothers	51	50	Mean QTc slightly longer in children of anti-Ro/SSA-positive mothers (*p* = 0.06)
Gerosa et al. (125)	Children of AD mothers	60	30	No difference in the prevalence of QTc prolongation between the groups
Bourrè-Tessier et al. (33)	Adult SLE patients (two studies)	57	93	5.1- to 12.6-times higher risk of QTc prolongation in anti-Ro/SSA-positive vs negative group
		113	165	
Lazzerini et al. (29)	Adult CTD patients	25 (SLE, 9; SS, 13; UCTD, 2; MCTD, 1)	24 (SLE, 13; SS, 3; UCTD, 6; MCTD, 2)	Mean QTc significantly longer and prevalence of QTc prolongation significantly higher in anti-Ro/SSA-positive subjects; significant correlation between anti-Ro/SSA-52kD concentration and QTc duration
Nomoura et al. (126)	Adult SLE patients	43	47	Anti-Ro/SSA positivity slightly more frequent among SLE patients with QTc prolongation (*p* = 0.08)
Alkmim Teixera et al. (35)	Adult SLE patients	111	206	No difference in the prevalence of marked QTc prolongation (>500 ms) between groups
Massie et al. (37)	Adult SSc patients	148	541	No difference in the prevalence of QTc prolongation between groups
Bourrè-Tessier et al. (34)	Adult SLE patients	283	314	Prevalence of QTc prolongation slightly higher in anti-Ro/SSA-positive subjects, but not significantly for wide confidence intervals
Pisoni et al. (30)	Adult AD patients	55 (SLE, 16; SS, 20; SSc, 3; UCTD, 11; MCTD, 1; PM/DM, 2; other ADs, 2)	18 (SLE, 14; SS, 1; UCTD, 1; other ADs, 1)	Anti-Ro/SSA positivity significantly more frequent among CTD patients with QTc prolongation (all patients with QTc prolongation were anti-Ro/SSA-positive)

*CTD, connective tissue disease; AD, autoimmune disease; SLE, systemic lupus erythematosus; SS, Sjögren’s syndrome; SSc, systemic sclerosis; UCTD, undifferentiated connective tissue disease; MCTD, mixed connective tissue disease; PM/DM, polymyositis/dermatomyositis*.

In addition to the above reported forthrightly supporting data, there are other studies that although not observing significant differences between anti-Ro/SSA-positive and negative patients in terms of mean QTc length and/or QTc prolongation prevalence, nevertheless found differences in these parameters that were very close to statistical significance. This is the case of the pediatric study of Motta et al. (124) (QTc of infants of anti-Ro/SSA-positive mothers slightly prolonged vs control group, *p* = 0.06), as well as of the adult studies of Gordon et al. (121) (QTc slightly longer in the anti-Ro/SSA-positive CTD group, *p* = 0.06), Nomura et al. (126) (anti-Ro/SSA positivity slightly more frequent among SLE patients with QTc prolongation, *p* = 0.08), and Bourrè-Tessier et al. (34) [proportion of SLE patients with prolonged QTc slightly higher in anti-Ro/SSA-52kD-positive group, although not reaching significance for wide confidence intervals].

Although the majority of the data point to an association between anti-Ro/SSA and QTc prolongation, there are conflicting results from other studies, either in children (123, 125) and adults (27, 35, 37). However, it should be noted that one of these studies (37) was performed in SSc patients, who frequently display anti-Ro/SSA but at a low level (127), thus possibly not high enough for the threshold level required for QTc prolongation manifestation (29, 128); in another one (35), involving SLE patients, the authors used a cutoff for QTc prolongation (>500 ms) probably not adequate to detect the phenomenon in this setting, as previous studies consistently demonstrated that in the large majority of the anti-Ro/SSA-positive CTD patients with QTc prolongation values ranged from 440 to 500 ms.

Besides anti-Ro/SSA, some lines of evidence suggest that other autoantibodies, i.e., anti-beta1-adrenergic receptor antibodies (anti-β1) and anti-voltage-gated potassium channel Kv1.4 antibodies (anti-Kv1.4), may be responsible of immuno-mediated forms of acquired LQTS.

Anti-β1 are frequently detected in idiopathic dilated cardiomyopathy (IDC, 30–50%), but also in Chagas’disease and in subjects with primary electrical disturbances (112). IDC is often complicated by ventricular arrhythmias [including TdP (129–134)] and SCD (135), with QT dynamicity representing an independent predictor of major arrhythmic events (136). Since the underlying mechanisms of such electrical instability are not fully clarified, and increasing evidence indicates that autoimmunity plays a relevant role in IDC pathogenesis (137), a possible link between anti-β1 and arrhythmic risk has been investigated. In IDC patients, circulating anti-β1 are associated with increased all-cause and cardiovascular mortality risk (138). Moreover, by studying 104 IDC subjects, Iwata et al. (139) demonstrated that the presence of anti-β1 independently predicted ventricular tachycardia and SCD. The following evidence in an animal model that induction of anti-β1 autoimmunity was concomitantly associated with QT/RR interval prolongation (with a parallel increase in APD at *ex vivo* electrophysiological examination), sustained ventricular tachycardia, and SCD (140) suggests that these antibodies may increase *in vivo* the risk of life-threatening arrhythmias at least in part by prolonging QTc.

Finally, an association of anti-Kv1.4 and LQTS has been demonstrated in patients affected with myasthenia gravis (MG), an autoimmune disease primarily affecting the neuromuscular function (141). The Kv1.4 protein is one of the forming α-subunits (Kv) of the voltage-gated potassium channel (VGKC), which plays a crucial role in the acetylcholine presynaptic release, but also in the cardiac repolarization (142). Indeed, Kv1.4 is also expressed in ventricular cardiomyocytes as pore-forming subunit of the channel responsible for the slowly recovering component of Ito, the main current of the phase 1 (early repolarization) of cardiac AP (143). Recent studies indicate that anti-Kv1.4 are relatively frequently detected in MG patients and their presence associates with QTc prolongation (~15–35% of positive cases) (144, 145). Moreover, in a cohort of 650 MG patients, Suzuki et al. (144) reported that among 70 anti-Kv1.4-positive subjects (14%), two died of lethal QT-associated arrhythmias (TdP in one case, SCD in a patient who had QTc prolongation in the other one). Notably, at least two further reports of TdP in MG patients are present in the literature (146, 147).

### Mechanisms

**Figure 3:**
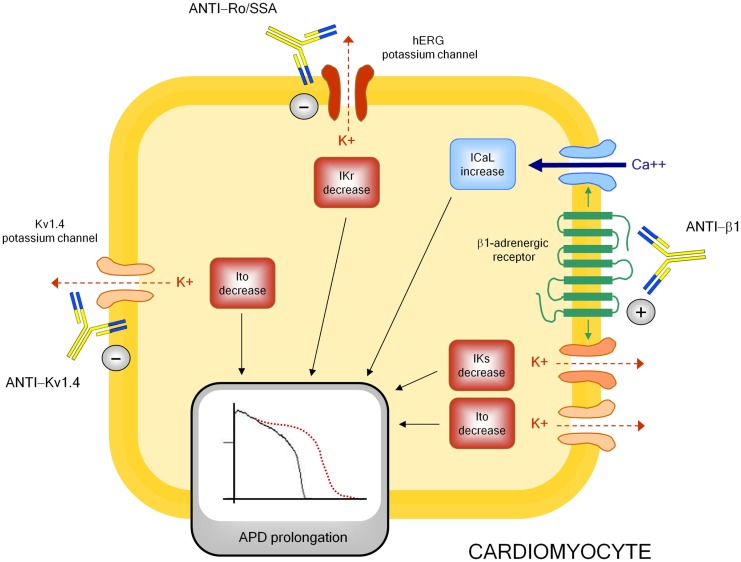
**Autoantibody-mediated QTc prolongation: molecular targets and electrophysiological consequences**. Anti-β1, anti β1-adrenergic receptor antibodies; Ito, transient outward potassium current; IKr, rapid component of the delayed rectifier current; IKs, slow component of the delayed rectifier current; ICaL, L-type calcium current; APD, action potential duration.

Although mechanisms underlying autoimmune-mediated LQTS are not fully known, accumulating evidence indicates that autoantibodies may directly affect cardiomyocyte electric properties by interfering on ion channels function (Figure 3).

The electrophysiological effects of anti-Ro/SSA are largely recognized, but mostly in the setting of congenital-AVB. Experimental studies clearly demonstrated the ability of anti-Ro/SSA from mothers with children with congenital-AVB in biochemically cross-reacting with l-type and T-type calcium-channels, thus significantly inhibiting the related currents (ICaL, ICaT), which both play a key role in the AP of heart conduction system cells (148). More in detail, it has been proven that anti-Ro/SSA specifically recognize the α_1_ pore-forming subunit of the calcium channels through a binding site localized on the extracellular loop of domain I S5–S6 (122, 149–153). The hypothesis is that Ro protein (particularly Ro52-kD) shares structural similarities with calcium channels, thus explaining a cross-reactivity of anti-Ro/SSA as a result of molecular mimicry mechanisms. Keeping this in mind, and in consideration of the fact that calcium and potassium channels belong to the same superfamily of voltage-gated ion channels in which, in particular, the structure of the voltage-sensor sequence is highly conserved (154), it is conceivable that a concomitant inhibitory cross-reactivity with potassium channels may be responsible of the effects of anti-Ro/SSA on QTc interval by impairing ventricular repolarization. In accordance with this view, some recent data suggest that hERG-potassium channel, conducting IKr, may represent a further specific target of anti-Ro/SSA. As already cited, Nakamura et al. (120) demonstrated that both serum and purified IgGs from an anti-Ro/SSA-positive woman with extreme QTc prolongation and TdPs specifically reacted with hERG-channel and induced a concentration-dependent and fully reversible inhibition of IKr. In a very recent study performed in the laboratory of Boutjdir in collaboration with our institution, these findings have been confirmed and expanded in a larger number of subjects, by comparing anti-Ro/SSA-positive vs negative CTD patients, as well as in an animal model. In particular, electrophysiological and biochemical evidence is provided that anti-Ro/SSA inhibit IKr and prolong APD by directly binding to the hERG-channel protein, likely at the pore region where homology with Ro-52kD antigen is present. Moreover, Ro-52kD antigen immunized guinea-pigs showed QTc prolongation on ECG after developing high titers of anti-Ro/SSA (155). In accordance with these results, strongly suggestive of a mechanism dependent on a purely electrophysiological interference on the heart, recent preliminary data from single case reports demonstrated the effectiveness of immunosuppressive therapy in reversing anti-Ro/SSA-associated electrocardiographic abnormalities *in vivo*, at least in adults (116, 156–158).

Despite this evidence, clinical studies analyzing the relationship between anti-Ro/SSA and QTc showed some degrees of discrepancy. Moreover, even among studies demonstrating significant association, markedly different percentages of QTc prolongation in anti-Ro/SSA-positive CTD patients were observed (~10–60%) (29). Although previously reported data (29, 30) suggest that it may be explained, at least in part, by differences among CTD cohorts in terms of autoantibody concentration and specificity [high levels of anti-Ro/SSA-52kD are particularly frequent in SS, much less in SLE and rarely in SSc (159)], or disease-related inflammatory burden (and thus cytokine levels), the above electrophysiological data, by indicating that anti-Ro/SSA inhibit both calcium and hERG-potassium currents, provide a further pathophysiological mechanism possibly contributing to differences observed. Indeed, it is well recognized that calcium and potassium channels have conflicting effects on APD, thus on QTc length. A block of the inward ICaL during the plateau phase shortens, while an inhibition of outward IKr during repolarization prolongs APD (160). Thus, it is conceivable that a concomitant inhibitory effect of anti-Ro/SSA on calcium channels can partially counteract the IKr inihibition-dependent APD prolonging effects *in vivo*, thus reducing the actual extent of QTc prolongation observed (128). In this view, intrinsic (inherited or acquired) differences in potassium and calcium channel expression on patient’s cardiomyocytes may participate in the QTc variability observed. In conclusion, evidence indicates that anti-Ro/SSA inhibit IKr, but the clinical phenotype may not be the same for each patient as a result of several modifying factors, including the anti-Ro/SSA level, the degree of systemic inflammation, and the peculiar cardiomyocyte ion channels’ profile.

A modulating activity on ion channel function seems to be also critically involved in the mechanism by which anti-β1 prolong APD and QTc, although in this case the effect is indirect via a stimulating interaction with the myocardial β1-adrenergic receptor. Indeed, some basic studies demonstrated that anti-β1 produced a profound electrical remodeling of the cardiomyocyte, mainly involving potassium and calcium conductance. Christ et al. (161) found that purified anti-β1, obtained from IDC patients, increased APD and ICaL in isolated rat and human cardiomyocytes. Later on, by analyzing isolated ventricular myocytes from rabbits immunized with a synthetic peptide corresponding to the second extracellular loop of β1-adrenergic receptors, Fukuda et al. (140) showed a significant decrease (~35–45%) of Ito1 and Iks. Moreover, they demonstrated APD prolongation and early afterdepolarization in the right ventricular papillary muscle, as well as a longer QT/RR interval ratio and a higher prevalence of sustained ventricular tachycardia in immunized vs control rabbits.

## Do Inflammation and Immunity Play a Role in Congenital LQTS?

Recent data intriguingly suggest that inflammation and immunity may be also involved in modulating the clinical expression of congenital LQTS, possibly triggering or enhancing electrical instability in patients already genetically predisposed to arrhythmias.

Rizzo et al. (162) performed a histopathologic study on stellate ganglia specimens obtained from 12 patients, 8 with different forms of congenital LQTS and 4 with catecholaminergic polymorphic ventricular tachycardia (CPVT), who underwent left cardiac sympathetic denervation for malignant intractable arrhythmias. Indeed, all the patients were severely sympthomatic before the ganglionectomy, with most patients having had multiple shocks from a previously implantable cardioverter defibrillator (including arrhythmic storms), and the procedure resulted in a rhythm stabilization in almost all the cases. Examination of patients’ stellate ganglia revealed low-grade but distinct inflammatory infiltrates composed by activated T lymphocytes and macrophages, indicative of a chronic T-cell-mediated ganglionitis. Moreover, morphometric analysis demonstrated that the number of T cells/mm^2^ were significantly higher in the ganglia of these patients when compared with those obtained from 10 sex- and age-matched control subjects accidentally died. On the basis of these findings, the authors speculated that a T-cell-mediated cytotoxicity toward ganglion cells may boost adrenergic activity through release of inflammatory mediators in ganglia and in this manner contribute to the electric instability in LQTS/CPVT patients, particularly in those who are heavily symptomatic. In accordance with this view, intracardiac ganglionitis and its pro-arrythmic potential have been previously described in LQTS patients who died suddenly, the first time over 35 years ago (163–165). Moreover, although the origin of inflammatory infiltrates remains unknown, Rizzo et al. (162) put forward the hypothesis of a viral (however not herpes-virus DNA was found in specimens) or autoimmune pathogenesis. As concerns this latter mechanism, Moss et al. (166) underlined how all patients had recurrent syncope and/or many defibrillator shocks, and both transient hypoperfusion and recurrent shocks could cause ganglionic cell injury with protein damage putatively resulting in a secondary autoimmune reaction with manifestations of ganglionitis. In any case, independently whether ganglionitis is a primary event or a phenomenon secondarily occurring after first severe arrhythmic episodes (thus triggering a self-aggravating loop), it is conceivable that it may have played a role in precipitating life-threatening tachyarrhythmias since stellectomy induced rhythm stabilization in almost all patients. These findings, although preliminary, intriguingly suggest that immuno-inflammatory pathways could in the future represent a novel target in the therapeutic approach to congenital LQTS, particularly in patients with intractable arrhythmias despite appropriate standard therapy.

Although these considerations primarily point to the therapeutic potential of interventions lowering the degree of the immuno-inflammatory response, a very recent study from the group of Nattel (167) suggests that a selective stimulation of the immune system may be also theoretically useful in the treatment of congenital LQTS. Starting from previous evidence demonstrating that in a subpopulation of IDC patients, autoantibodies against the KCNQ1-encoded Kv7.1 potassium channel were associated with QTc shortening possibly by increasing IKs conductance (168). Li et al. (167) immunized rabbits with KCNQ1-channel peptide, thus inducing high circulating anti-KCNQ1 antibody titers. As expected, these animals developed significant QTc shortening compared to controls, as well as APD decrease and IKs densities enhancement in left ventricular cardiomyocytes when isolated. Since these findings indicate that KCNQ1 autoimmunity accelerates cardiac repolarization by increasing channel function, the potential consequences of this immunization were tested in a well-recognized rabbit model of human LQTS, induced by infusion of methoxamine and dofetilide. KCNQ1-immunized animals showed much less striking ECG changes with significantly less severe QTc prolongation, compared to sham-immunized animals upon drug challenge (17.5 vs 73.4% increase). Moreover, life-threatening ventricular arrhythmias, particularly TdP, were observed in the sham-group only. On the basis of these results, the authors speculated that by enhancing repolarization reserve KCNQ1 vaccination may be therapeutically useful in patients with congenital LQTS resistant to conventional treatments, thus opening new exciting avenues in antiarrhythmic therapy (167).

## Conclusion and Perspectives

In the latest years, inflammation and immunity have been increasingly recognized as novel factors crucially involved in modulating arrhythmic risk, an effect in part resulting from a significant impact on ventricular repolarization.

A number of considerations suggest that these phenomena may have relevant clinical implications, also in terms of therapeutic perspectives.

First, although inflammatory and autoimmune mechanisms are in most cases probably not *per se* able to induce a QTc prolongation as critical as to induce TdP [but actually this is true for all recognized causes of LQTS, when present alone (130, 169)], nevertheless they can reduce the ventricular repolarization reserve, thereby significantly increasing the risk of life-threatening arrhythmias in the presence of other classical QT-prolonging factors (drugs, electrolyte imbalances, genetic polymorphisms, etc.). While it is well conceivable that these events may take place in patients with autoimmune chronic inflammatory diseases, thus putatively contributing to explain the increased risk of sudden death observed in the course of RA (64) and CTDs (170), nevertheless asymptomatic low-grade chronic inflammation and/or circulating anti-Ro/SSA may be also silently involved, as a predisposing factor, in a number of unexpected life-threatening arrhythmias, including drug-induced TdP, and SCDs occurring in general population.

**Figure 4:**
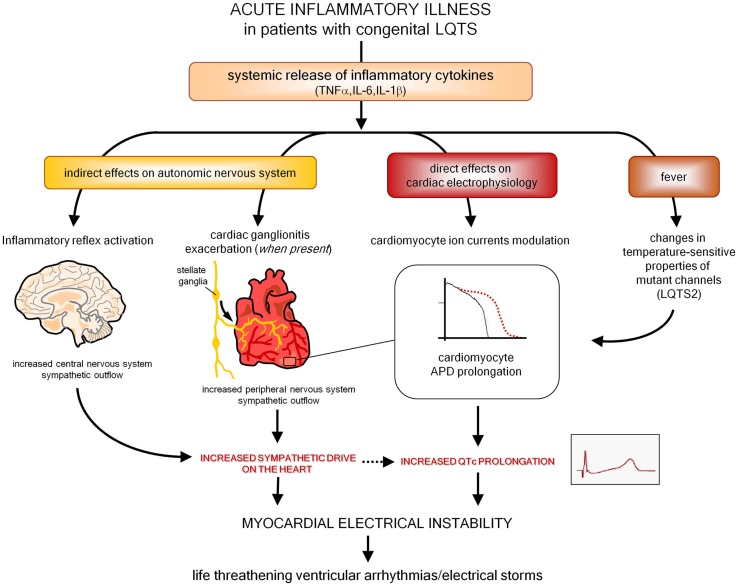
**Putative pathways involved in exacerbating myocardial electrical instability in patients with congenital LQTS during an acute inflammatory illness**. LQTS, long QT syndrome; APD, action potential duration.

Moreover, as also preliminarily suggested by a recent histopathology study in stellate ganglia (161), it is also probable that inflammation and immunity may enhance arrhythmic risk in patients with congenital LQTS. Indeed, it should be underlined that an acute inflammatory illness is recently recognized among the possible precipitant factors of malignant arrhythmias and electrical storms in these subjects (171–173). Although it has been demonstrated that fever has *per se* a role by influencing temperature-sensitive biophysical properties of mutant channels (particularly in LQTS2) (173, 174), it can also be speculated that in patients with congenital LQTS episodes of systemic inflammation may further increase arrhythmias susceptibility due to circulating cytokines directly affecting cardiomyocyte APD, and indirectly increasing sympathetic output from central and peripheral ANS (Figure 4). In this view, it is also conceivable that some acquired LQTS patients are occult (latent) carriers of mutations in LQTS-susceptibility genes that are unmasked under inflammatory/autoimmune conditions, with a potential different impact of immunotherapies on QTc.

Thus, in terms of therapeutic perspectives, in patients with inflammatory/autoimmune disease-associated QTc prolongation, besides avoiding any further acquired factor potentially prolonging QTc, and carefully balancing *pro* and *contra* when introducing any QT-prolonging drug, available data highlight the importance of minimizing systemic immuno-inflammatory burden through a tight control of disease activity, a goal now more feasible after the introduction of potent biologic therapies targeting the immune system.

As concerns QTc prolongation occurring in general population subjects displaying chronic low-grade systemic inflammation and/or specific autoantibodies in the absence of a clinically evident inflammatory/autoimmune disease, as well as malignant intractable arrhythmias in congenital LQTS patients with signs of immuno-inflammatory activation, no data are currently available on the therapeutic role of anti-inflammatory or immunomodulatory interventions. Nevertheless, accumulating evidence reviewed in this paper underlines the need for further specific investigations on this topic.

In conclusion, the potential impact of inflammatory and immunologic mechanisms on ventricular repolarization should be always carefully kept in mind, not only in the presence of a manifest immune-inflammatory disease, but also in subjects with QTc prolongation of unclear origin, or in patients with an already recognized LQTS (inherited or acquired) as a possible trigger for electrical instability. In this view, targeting immuno-inflammatory pathways may represent an attractive and innovative therapeutic approach in a number of LQTS patients.

## Conflict of Interest Statement

We do not have any financial support or other benefits from commercial sources for the work reported on in the manuscript, or any other financial interests which could create a potential conflict of interest or the appearance of a conflict of interest with regard to the work.
